# Supplementary material for: The IL-17A/IL-17RA Axis Is Not Related to Overall Survival and Cancer Stem Cell Modulation in Pancreatic Cancer
Source: Int J Mol Sci. 2020 Mar 23;21(6):2215. doi: 10.3390/ijms21062215 (PMC7139783; doi:10.3390/ijms21062215)
Supplement: Supplementary file 1 [file ijms-21-02215-s001.pdf]

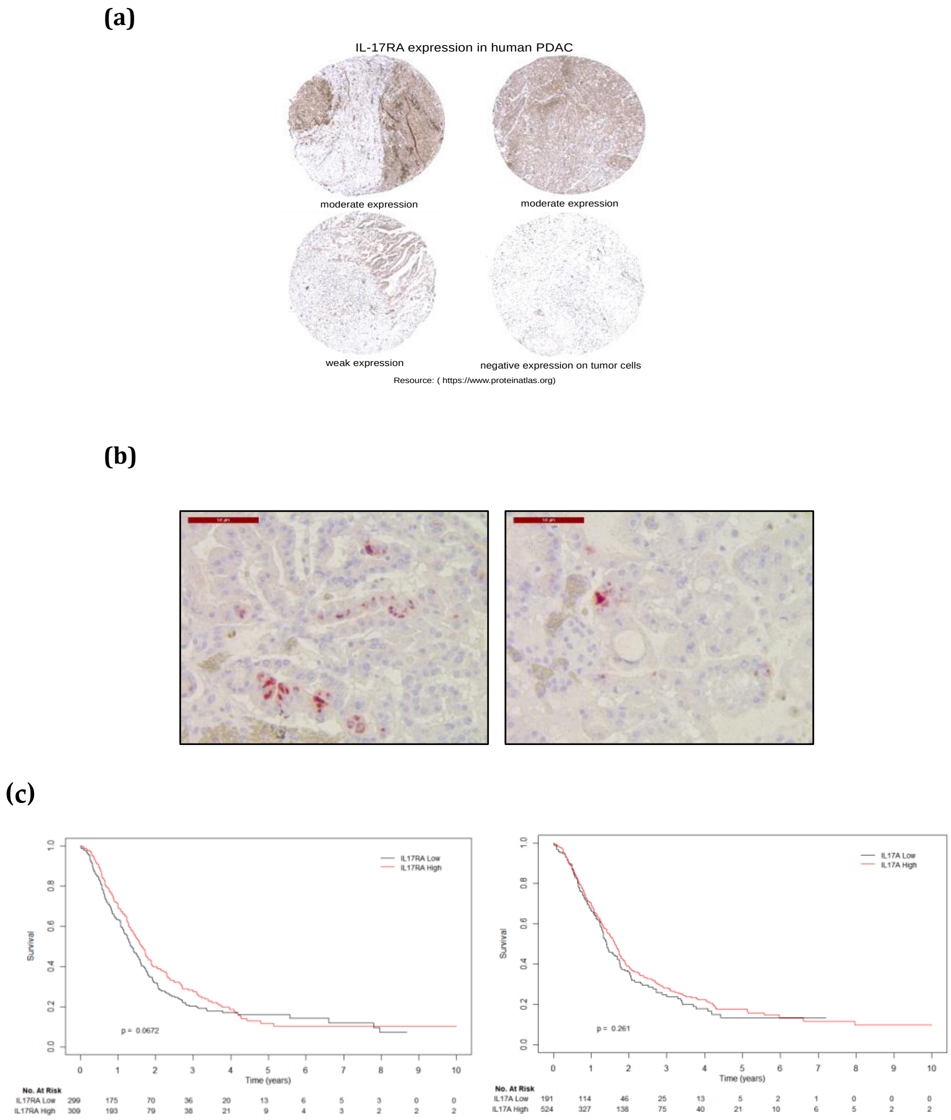

Figure S1. IL-17RA expression in human and mouse PDAC. (a) Publicly available PDAC samples showed a weak to moderate expression of IL-17RA (www.proteinatlas.org). (b) Pancreatic cancer cells express IL-17RA in a mouse model of pancreatic cancer. (c) Survival analysis of IL-17RA and IL-17A expression retrieved from the publicly available transcriptomic data of 903 patients with PDAC. There is no statistical significance between the survival of low (< 33rd percentile) and high (> 66th percentile) expression tumors regarding these two markers.
